# Clinical and mutation analysis of 51 probands with anophthalmia and/or severe microphthalmia from a single center

**DOI:** 10.1002/mgg3.2

**Published:** 2013-03-27

**Authors:** Christina Gerth-Kahlert, Kathleen Williamson, Morad Ansari, Jacqueline K Rainger, Volker Hingst, Theodor Zimmermann, Stefani Tech, Rudolf F Guthoff, Veronica van Heyningen, David R FitzPatrick

**Affiliations:** 1 Department of Ophthalmology, University of RostockGermany; 2 MRC Human Genetics Unit, MRC Institute of Genetics and Molecular Medicine at the University of Edinburgh, Western General HospitalEdinburgh, EH4 2XU, United Kingdom; 3 Department of Radiology, University of RostockGermany; 4 Department of Pediatrics, University of ErlangenGermany

**Keywords:** Anophthalmia, array CGH, *BMP4*, coloboma, de novo mutations, *FOXE3*, gene deletion, haploinsufficiency, microphthalmia, missense mutations, *OTX2*, *PAX6*, *RAX*, *SMOC1*, *SOX2*, *STRA6*, transcription factors

## Abstract

Clinical evaluation and mutation analysis was performed in 51 consecutive probands with severe eye malformations – anophthalmia and/or severe microphthalmia – seen in a single specialist ophthalmology center. The mutation analysis consisted of bidirectional sequencing of the coding regions of *SOX2*, *OTX2*, *PAX6* (paired domain), *STRA6*, *BMP4*, *SMOC1*, *FOXE3*, and *RAX*, and genome-wide array-based copy number assessment. Fifteen (29.4%) of the 51 probands had likely causative mutations affecting *SOX2* (9/51), *OTX2* (5/51), and *STRA6* (1/51). Of the cases with bilateral anophthalmia, 9/12 (75%) were found to be mutation positive. Three of these mutations were large genomic deletions encompassing *SOX2* (one case) or *OTX2* (two cases). Familial inheritance of three intragenic, plausibly pathogenic, and heterozygous mutations was observed. An unaffected carrier parent of an affected child with an identified *OTX2* mutation confirmed the previously reported nonpenetrance for this disorder. Two families with *SOX2* mutations demonstrated a parent and child both with significant but highly variable eye malformations. Heterozygous loss-of-function mutations in *SOX2* and *OTX2* are the most common genetic pathology associated with severe eye malformations and bi-allelic loss-of-function in *STRA6* is confirmed as an emerging cause of nonsyndromal eye malformations.

## Introduction

Severe eye malformations are a clinically important but rare group of developmental disorders with a combined live birth prevalence of 1 per 10,000 (Shah et al. 2011, 2012). Anophthalmia is the absence of the globe in presence of ocular adnexa. An extremely rare subgroup is termed true anophthalmia, which is associated with the complete absence of neuroectodermal tissue in the orbit. The more common presentation is termed “clinical anophthalmia.” In this form, no ocular tissue is visible on examination (Duke-Elder 1964) but remnants of the optic vesicle, muscle or optic nerve tissue or cysts are detected by orbital magnetic resonance imaging (MRI) or ultrasonography (for an example see Fig. 3). Microphthalmia can be defined as an eye with an axial length of <19 mm in a 1-year-old child or <21 mm in an adult (2 SD below normal; Weiss et al. 1989a,b). Microphthalmia can occur without (simple type) or with anterior and or posterior segment dysgenesis (complex type). Ocular coloboma, complex microphthalmia, and both clinical and true anophthalmia probably present a continuum of malformation severity.

Anophthalmia and complex microphthalmia are etiologically heterogeneous. Causative mutations are identified in many different genes including *SOX2* (Fantes et al. 2003), *OTX2* (Ragge et al. 2005a), *PAX6* (Glaser et al. 1994), *STRA6* (Pasutto et al. 2007), *FOXE3* (Reis et al. 2010), *RAX* (Voronina et al. 2004; Lequeux et al. 2008), *SMOC1* (Abouzeid et al. 2011; Okada et al. 2011; Rainger et al. 2011), and *BMP4* (Bakrania et al. 2008). Heterozygous loss-of-function mutations in either *SOX2* account for about 15% of cases with severe bilateral eye developmental defects (Bardakjian et al. 1993; FitzPatrick 1993; Ragge et al. 2005b) or *OTX2* mutations ranging between 2% and 8% of the investigated cohort (Ragge et al. 2005a; Wyatt et al. 2008; Schilter et al. 2011). Heterozygous, loss-of-function mutations in the *OTX2* gene commonly occur de novo in affected children but in 35% of cases the mutation is inherited from a completely normal parent (Ragge et al. 2005a; Schilter et al. 2011). *SOX2* mutations almost always occur de novo and appear to be fully penetrant with all reports of sibling recurrence associated with detectable mosaicism in the mother (Faivre et al. 2006; Chassaing et al. 2007; Schneider et al. 2008).

Here, we describe a clinical evaluation and comprehensive mutation analysis in 51 probands with microphthalmia and/or anophthalmia seen in a single specialist ophthalmology center. Genome-wide oligonucleotide array-based comparative genomic hybridization (aCGH) was performed on each proband together with sequencing of the coding regions of *SOX2*, *OTX2*, *PAX6* (paired domain), *STRA6*, *BMP4*, *SMOC1*, *FOXE3*, and *RAX*. We identified different mutations in the 15/51 (29.4%) of the probands in *SOX2* (9/51), *OTX2* (5/51), and *STRA6* (1/51). The mutation-positive individuals were more likely to have bilateral rather than unilateral eye disease (13:3). Indeed 9/12 (75%) of the cases with bilateral anophthalmia (BA) were found to be mutation positive. Familial inheritance of a plausibly pathogenic heterozygous mutation was seen for both *SOX2* (two families) and *OTX2* (one family). In the latter family, an unaffected carrier parent of an affected child with an identified *OTX2* mutation was consistent with the previously reported nonpenetrance for this disorder. For *SOX2*, both families demonstrated full penetrance but variable expressivity with parents and children associated with *SOX2* mutation each having significant eye malformations.

## Methods

### Case ascertainment

Patients and parents of children with unilateral anophtalmia (UA) or BA or microphthalmia referred to the Department of Ophthalmology at the University of Rostock for evaluation of surgical therapy with orbital or socket expander were invited to participate in this study. The study was approved by the Ethics board of the University of Rostock (reference A 2008-62) and the U.K. Multiregional Ethics Committee (Reference: 06/MRE00/76).

### Phenotype assessment

All patients and available parents received a comprehensive eye examination including dilated fundus examination. Birth history, extraocular anomalies, and developmental data with emphasis on speech and motor development were extracted from medical reports and parent interview. Sleep abnormalities were evaluated using the sleep disturbance scale for children through parent interviewing (Eitner et al. 2007). Z-scores for height, weight, and head circumference were calculated using the LMS method based on data from U.K. children (Cole et al. 1998). Cerebral MRI was reassessed if available. The analysis of intracerebral abnormalities was focused on corpus callosum, hypothalamus, and hippocampus integrity based on published data of patients with microphthalmia or anophthalmia (Leichtman et al. 1994; Warburg et al. 1997; Arnold et al. 2003; Ragge et al. 2005a,b; Makhoul et al. 2007; Kelberman et al. 2008; Schneider et al. 2008; Tajima et al. 2009). Agenesis of the corpus callosum refers to complete absence whereas hypoplasia refers to a fully formed corpus callosum that is thinner than expected for age. Results of previous image evaluation were included in the study if original MRIs were not available.

### Intragenic mutation analysis

Genomic DNA was whole genome amplified using GenomiPhi V2 DNA Amplification Kit (GE Healthcare Life Sciences, Pittsburg, PA) prior to mutation screening. To ensure a complete dataset, all eight genes were screened in all affected individuals regardless of whether a plausibly pathogenic mutation had been detected. Identified mutations were validated using genomic DNA that had not been whole genome amplified. For each gene, the amplicons encompassing the coding exons and the intronic splice junctions were designed using ExonPrimer or by manual means. Full methods and oligonucleotide sequences are available on request. Each amplicon was sequenced on the ABI 3730 DNA Analyzer (Applied Biosystems, Foster City, CA) and the results were analyzed with Mutation Surveyor v3.30 (SoftGenetics, State College, PA). The genomic sequence identifiers for each gene analyzed are as follows: *SOX2*, NT_005612.15 GI:88966845; *OTX2*, NT_026437.11 GI:51493278; *PAX6*, NC_000011.9 GI:224589802; *STRA6*, NG_009207.1 GI:219275560; *BMP4*, NG_009215.1 GI:219521814; *SMOC1*, NC_000014.8 GI:224589805; *FOXE3*, NG_016192.1 GI:281182531; and *RAX*, NG_013031.1 GI:260655998.

### In silico analysis

Assessment of the pathogenic potential of the missense mutations used different bioinformatics approaches. PolyPhen-2 (http://genetics.bwh.harvard.edu/pph2/), SIFT (http://sift.jcvi.org), and ALIGN-GVGD (http://agvgd.iarc.fr/agvgd_input.php) use different algorithms to produce a combined score based on a multispecies alignment and the biophysical characteristics of the altered amino acids (Tavtigian et al. 2006; Kumar et al. 2009; Adzhubei et al. 2010). Mutation Taster (http://www.mutationtaster.org/) uses a Bayes Classifier that was trained on large sets of pathological and benign amino acid substitutions. The possible effect of one of the missense mutation on splice site function used different prediction algorithms on the mutant and wild-type allele implemented online using SSF (http://www.umd.be/searchSpliceSite.html), MaxEnt (Yeo and Burge 2004), and HSF (http://www.umd.be/HSF/). These algorithms were implemented via the Alamut package (Interactive Biosoftware, San Diego, CA). The haploinsufficiency (HI) score was calculated manually using the BED format dataset from the original report (Huang et al. 2010) loaded as a custom track into the UCSC Genome Browser (http://genome.ucsc.edu/).

### Array-based comparative genomic hybridization

Genome-wide analysis of DNA copy number aberrations was carried out using the Roche Nimblegen 12X135k whole-genome array (median probe spacing of approximately 12 kb) according to the manufacturer's instructions, modified as follows: 250–800 ng of genomic DNA from patient and sex-matched control samples (pool of five male or female samples) was labeled overnight followed by hybridization for 72 h. After washing, slides were scanned using the Roche MS200 scanner, and analyzed using the software NimbleScan (Roche Nimblegen, Madison, WI). The CGH-segMNT module of NimbleScan was used for the analysis with a minimum segment length of five probes and an averaging window of 130 kb. Results were compared with the Database of Genomic Variants (http://projects.tcag.ca/variations) and polymorphic copy number variation excluded. Deletions of the *OTX2* gene were confirmed using the Roche Nimblegen 3X720k whole-genome array (median probe spacing of approximately 2.5 kb) as described above, with an averaging window of 20 kb.

### Fluorescence in situ hybridization

Deletion of the *SOX2* gene was confirmed by fluorescence in situ hybridization (FISH). Metaphase chromosomes were prepared from the patient lymphocytes as described elsewhere (Weier et al. 1991). The BAC clone RP11-43F17, which spans *SOX2*, was selected from the UCSC Human Genome Browser (http://genome.ucsc.edu) and the probe was labeled with biotin-16-dUTP (Roche, Basel, Switzerland) by nick translation (Fantes et al., 2008). Following hybridization, slides were mounted with a drop of Vectorshield antifadent containing DAPI (Sigma Aldrich, St Louis, MO). Antibody detection was carried out by fluorescent microscopy using a Zeiss Axioscop microscope. Images were collected using a cooled CCD (charged coupled device) camera (Smart-Capture software).

## Results

### Summary of cohort and mutation analysis results

Fifty-one unrelated probands were enrolled in the study. Inclusion of mutation-positive parents gives a total of 54 individuals (Table 1). One of these carrier parents did not show any ocular abnormalities but is included as a “case.” The male:female ratio was 24:30. Of the 53 affected cases, 42 had anophthalmia (unilateral:bilateral = 30:12), 10 had severe microphthalmia (unilateral:bilateral = 7:3), one (a mutation-positive parent) had bilateral coloboma.

**Table 1:** Phenotype comparison of patients with and without identified mutations

	No mutation identified	*OTX2* mutation	*SOX2* mutation	*STRA6* mutation	Total	Molecular diagnostic rate
Cases (families)	36 (36)	6 (5)	11 (9)	1 (1)	54 (51)	33.3 (29.4)
Male:Female	16:20	2:4	5:6	1:0	24:30	33.3:33.3
Ocular phenotype						
Bilateral anophthalmia	3	3	5	1	12	75.0
Unilateral anophthalmia	23	2	5^2^	0	30	23.3
Bilateral microphthalmia	3	0	0	0	3	0.0
Unilateral microphthalmia	7	0	0	0	7	0.0
Bilateral iris/chorio-retinal coloboma	0	0	1^2^	0	1^2^	
Unaffected	0	1^2^	0	0	1^2^	
No light perception in both eyes^1^	6	4	7	1	18	66.7
Extraocular abnormalities						
Intracerebral MRI (excluding optic tract)	11/21^3^	1/5	6/8	0/1		
Facial clefts	6	0	0	0		
Extremities	3	1	0	0		
Ear abnormalities	5	1	0	0		
Heart	5	0	0	0		
Kidney	3	0	0	0		

MRI, magnetic resonance imaging.

1 At most recent visit.

2 One parent (mother) affected.

3 Available data.

No plausibly pathogenic mutations or copy number variants were identified in 36 cases. Of the 18 mutation-positive cases, one had bi-allelic intragenic compound heterozygous loss-of-function mutations in *STRA6*. Eleven cases from nine families had heterozygous loss-of-functions mutations affecting *SOX2*. Of these nine mutations, two were transmitted from mother to child. In both these families, analysis of the grandparental samples confirmed that the mutation had occurred de novo in the mother. Of the seven remaining *SOX2* mutations, three occurred de novo and four families did not have samples from both parents available for analysis (Table 2).

**Table 2:** Phenotype in patients with mutation in *SOX2* gene

FamID	3432				3194		2813		3171		3227				3303		3370		2850		3797	
									
CaseID	3432		3433		3194		2813		3171		3227		3228		3303		3370		2850		3797	
Nucleotide change	c.70_89del20		c.70_89del20		c.138_140dupTGC		c.244_245delTT		c.302A>G		c.368A>G		c.368A>G		c.479_480dupAC		c.841_851del11insA		1.6 Mb del		c277G>T	
Predicted protein change or genomic coordinates	p.(Asn24Argfs*65)				p.(Ala47dup)		p.(Leu82Valfs*13)		p.(His101Arg)		p.(Asp123Gly)				p. (Ala161Thrfs*4)		p. (Ala281Argfs*87)		chr3:182,649,000–184,339,000 (hg18)		p.(Glu93*)	
Paternal/maternal genotype	?/het (affected)		wt/wt		wt/wt		? (unavailable)/wt		? (unavailable)/wt		wt/het (affected)		wt/wt		wt/wt		? (unavailable)/wt		wt/wt		?/wt	
Ancestral background	German		German		German		German		Croatian		Austrian		Austrian		German		German		Kirgigistan/Sachalin		German	
Sex	Male		Female		Male		Male		Male		Female		Female		Female		Male		Female		Female	
Gestational age (weeks+days)	37		41		42		38 + 6				40		40		38		32 + 5		40 + 4		39 + 4	
Birth weight (g) [*Z*-score]	3700 [0.3]		3830 [0.91]		3440 [−0.32]		2950 [−1.27]				3250 [−0.34]		3050 [−0.79]		3090 [−0.7]		2000 [−0.18]		3840 [0.94]		4210 [1.7]	
Birth occipitofrontal circumference (cm) [*Z*-score]	34.5 [−0.55]				33.7 [−1.18]		31.5 [−2.9]				34 [−0.44]				32 [−2.06]				34 [−0.44]		35 [0.37]	
Height (cm) [*Z*-score]	101 [−0.36]		178 [2.35]		110 [−1.2]		80 [−0.62]		70 [1.93]		85 [0.28]		167 [0.53]		110 [−2.1]		80 [−4.7]		120 [−1.8]		78 [−0.91]	
Weight (kg) [Z-score]	18		57 [−1.97]		19 [−0.72]		10 [−1.27]		8.8 [1.43]		10.9 [−0.58]		59 [−1.64]		20 [−0.96]		10 [−4.04]		23 [−1.1]		10 [−0.75]	
Age at growth measurement	4 years		28.5 years		6 years		1.5 years		5 months		1.9 years		34 years		7 years		3.4 years		8.5 years		1.5 years	
Occipitofrontal circumference (cm) at age (years) [*Z*-score]	51 at 4 [−0.083]				45.5 at 4 [−4.5]		47.8 at 3.8 [−2.9]				48 at 3 [−1.9]				45.6 at 2 [−2.8]		46.0 at 5 [−4.4]		49 at 8.9 [−3.4]		44 at 1.5 [−3.36]	
Age at last assessment	3.0 years		27.5 years		2 years		1.5 years		5 months		1.7 years		34 years		6 years		2.4 years		8.5 years		3 months	

Ocular phenotype	RE	LE	RE	LE	RE	LE	RE	LE	RE	LE	RE	LE	RE	LE	RE	LE	RE	LE	RE	LE	RE	LE
Clinical anophthalmia	x			x	x	x	x	x	x	x	x				x		x	x	x	x	x	
Microphthalmia (axial length, mm)												x (18.5)				x						x (16.3 mm)
Microcornea (diameter, mm)												x (8.5)		x		x						x (4 × 7)
Sclerocornea																x						x
Coloboma												Retinal/choroidal	Iris/retinal/choroidal	Iris/retinal/choroidal								
No abnormalities		x	x																			
Vision (decimal)	No	Fixation	0.7	No	No	No	No	No	No	No	No	No fixation	1.0	0.025	No	No	No	No	No	No	No	LP

Extraocular abnormalities																						
Cerebral MRI (at age)	Cavum vergae (2.9 years)		Not done		Small posterior corpus callosum, septum pellucidum cyst (3 months)		Fronto-temporal cerebral volume reduction (3 months)		Small septum pellucidum cyst (2 months)		Normal (10 days)		Not done		Frontal cerebral volume reduction (5.8 years)		Not done		Pineal cyst (4.5 years)		Normal (1 month)	
Hearing	Normal? (OAEs at age 3 negative but had Eustachian catarrh)		No test available		OAEs at 6 years normal		OAEs at birth and age 4 years normal		No test available		Normal		Normal		OAEs after birth abnormal		OAEs at birth normal		OAEs at birth normal		Normal	
Speech development	Delayed		Reduced		Good		Severe delayed (baby babble)				Normal		Normal		Severe delayed (only mommy, daddy)		Severe delayed (baby babble)		Normal		Normal	
Motor development	Delayed		Spastic gait		Walked at 2 years		Walks with support				Delayed		Normal		Severe delayed/cannot sit or walk		Severe delayed/cannot sit or walk		Normal		Normal	
Sleep	Normal		Normal		Normal		Wakes up at night irregularly				Normal		Normal		Normal		Normal		Normal		Normal	

The genomic sequence identifiers for *SOX2* are NT_005612.15 GI:88966845. het, heterozygous; LE, left eye; MRI, magnetic resonance imaging; OAEs, otoacustic emissions; RE, right eye; wt, wild-type; ?, unknown. ?, unknown; het, heterozygous; wt, wild-type.

Six cases from five families had heterozygous loss-of-function mutations affecting *OTX2*. One of these mutations was transmitted from mother to child. One mutation occurred de novo in the affected individual and in the remaining three families samples from both parents were not available to determine inherited status. None of the mutation-positive cases has associated facial clefts, heart, or kidney anomalies (Table 3).

**Table 3:** Phenotype in patients with *OTX2* mutation

FamID	2896		3197		2867				3346		3000	
						
CaseID	2896		3197		2867		3362		3346		3000	
Nucleotide change	c.234delC		c.249G>T		c.276_294del19		c.276_294del19		455 kb deletion		6.5 Mb deletion	
Predicted protein change	p.(Glu79Serfs*30)		p.(Gln83His) or p.?		p.(Lys92Asnfs*11)				chr14:56,224,000-56,679,000 (hg18)		chr14:56,094,000-62,594,000 (hg18)	
Paternal/maternal genotype	?/wt		wt/?		wt/het (unaffected)		?/?		wt/wt		?/?	
Ancestral background	German		Arabic Emirates		German		German		Polish		German	
Sex	Male		Female		Female		Female		Male		Female	
Gestational age (weeks + days)	41 + 5				36				39 + 2		39	
Birth weight (g) [*Z*-score]	3870 [0.31]				2400 [−0.55]				2920 [−0.98]		2810 [−1.01]	
Birth occipitofrontal circumference (cm) [*Z*-score]	34 [−0.95]				33 [−1.2]				38 [2.2]			
Height (cm) [*Z*-score]	98 [−3.6]		142 [2.19]		113 [0.93]				95 [0.9]		112 [−1.7]	
Weight (kg) [*Z*-score]	13 [−4.3]		39 [1.8]		13 [−2.8]				12.5 [−0.88]		19 [−1.3]	
Age at growth measurement (years)	6		8.6		5				2.6		7	
Occipitofrontal circumference (cm) at age (years) [*Z*-score]	48.9 at 5.7 [−2.7]		No microcephaly		47.7 at 5 [−3.3]				49 at 3 [−1.7]		47 at 8.8 [−5.1]	
Age at last assessment (years)	6		8.6		6		34		1		8.7	
Ocular phenotype	RE	LE	RE	LE	RE	LE	RE	LE	RE	LE	RE	LE
Clinical anophthalmia	x	x	x		x				x	x	x	x
Microphthalmia (axial length, mm)						x (15.0)						
Microcornea (diameter, mm)						x (8.0)						
Sclerocornea												
Coloboma						Retinal						
Other						No iris visible, dysplastic retina		Amblyopia				
No abnormalities				x			x					
Vision (decimal)	No	No	No	1.0	No	No	1.0	0.2	No	No	No	No
Extraocular abnormalities												
Cerebral MRI (at age)	No tractus opticus (3 months)		Normal (6 months)		Normal (3 months)		Not done		Normal (1 day)		Small pituitary gland (4.7 years)	
Hearing	Right OAE at birth normal		Normal		Normal				ABR and OAEs at 8 months normal		BERA at age 2.5 years: good responses from 60 to 30 db	
Extremities					Talipes equinovarus							
Ear malformation	Left microtia II°											
Speech development	Severe delayed (baby babble)		Normal		Delayed		Normal		Normal		Delayed	
Motor development	Severe delayed, does not crawl/walk		Normal		Delayed (walks short distances w/o support		Normal		Normal		Delayed	
Sleep	Sleep disorders (abnormal SDSC)		Normal		Sleep disorders (abnormal SDSC)		Normal		Normal		Wakes up at night	

The genomic sequence identifiers for *OTX2* are NT_026437.11 GI:51493278. ABR, auditory brainstem response; BERA, brainstem evoked response audiometry; LE, left eye; MRI, magnetic resonance imaging; OAEs, otoacustic emissions; RE, right eye; SDSC, sleep disturbance scale for children.

#### Segmental aneuploidy cases

**Figure 1:**
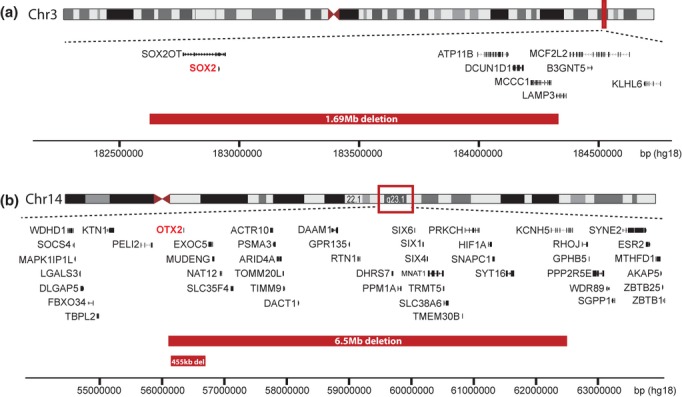
Schematic diagram showing three heterozygous deletions identified by array comparative genomic hybridization (CGH). (A) A de novo 1.69 Mb deletion in CaseID 2850 (chr3:182,649,000–184,339,000 equivalent to hg19 chr3:181,166,306–182,856,306) resulting in a loss of a single copy of *SOX2*, shown in red. Deletions are marked by red bars. Genomic coordinates are based on the March 2006 Human Genome Assembly (NCBI36/hg18). (B) A 6.5 Mb deletion in CaseID 3000 (chr14:56,094,000–62,594,000; equivalent to hg19 chr14:57,024,247–63,524-247) and a de novo 455 kb deletion in CaseID 3346 (chr14:56,224,000–56,679,000; equivalent to hg19 chr14:57,154,247–57,609,247). Both deletions resulted in a loss of a single copy of *OTX2*, shown in red.

*SOX2* HI in CaseID 2850 resulted from a de novo 1.6Mb genomic deletion (chr3:181,166,306–182,856,306; hg19) (Fig. 1). This deletion encompasses six genes in addition to *SOX2* (including *SOX2OT*). This child has BA but surprisingly had normal development with no significant extraocular features apart from a pineal cyst detected on MRI at 4.5 years (Table 2). This suggests that the other deleted genes have little or no haploinsufficient effect. The HI score is designed to predict a haploinsufficient effect of an individual gene. It is a combined metric incorporating evolutionary conservation of the open reading frame (ORF) and promoter, embryonic expression and proximity to known haploinsufficient genes in protein–protein and probabilistic gene interaction networks (Huang et al. 2010). The mean HI score of the genes in the deletion in CaseID 2850 is 35.5% and ranges from 4.6%.(*SOX2*) to 69.7% (*LAMP3*).

This was due to a deletion in two other affected individuals with *OTX2* heterozygous loss-of-function (Fig. 1). CaseID 3346 has a de novo 455Kb deletion (chr14:57,154,247–57,609,247; hg19) which encompasses only *OTX2* and the noncoding *OTX2-AS1* gene. This child is developing well with normal speech (he started to speak at age 8 months, loves to sing) and motor development. Now at age 2.6 years, he walks in areas known to him without any problems, even up and down stairs. His otoacoustic emissions (OAEs) and auditory brainstem responses (ABR) at age 8 months indicate a normal hearing level in both ears (Table 3). CaseID 3000 has a 6.5 Mb deletion (chr14:57,024,247–63,524,247; hg19), however, both parental samples were not available to determine de novo status (Fig. 1). This deletion encompassed 36 genes in addition to *OTX2,* including the known disease genes *SIX6* (MIM# 606326 Microphthalmia with cataract 2 – discussed below), *SIX1* (MIM# 601205 Brachiootic syndrome 3 and deafness, autosomal dominant 23), and *PRKCH* (MIM# 605437 susceptibility to cerebral infarction). The mean HI score of these genes is 40.5% and ranges from 0.5% (*PPM1A*) to 98.2% (*C14orf101*). This individual had BA with significant developmental delay (Table 3). Brainstem auditory evoked response (BAER) recording at age 2 weeks and 3 months revealed normal responses between 80 and 60 dB. No reliable response could be documented at 50 dB. She wore hearing aids till age 2 then she refused to wear them. Repeated BAER recordings at age 2.5 years showed good responses from 60 to 30 dB, which was confirmed at later age. The aCGH and FISH data for each of the whole gene deletion mutations are shown in Figure S1.

### *SOX2* mutation cases

#### Consequence of mutations

Of the eight intragenic mutations in *SOX2*, five would plausibly result in complete loss-of-function, four frameshift mutations, and one nonsense mutation (Tables 2 and 4). The remaining three mutations consisted of two missense changes, p.(His101Arg) and p.(Asp123Gly), and one in-frame duplication, p.(Ala47dup). Residues Ala47 and His101 lie within the HMG domain of *SOX2* and both are completely evolutionarily conserved to *Drosophila melanogaster* SoxNeuro protein. The HMG domain is known to make direct contact with DNA, to bend the DNA strand, and to mediate protein–protein interactions with the cooperative binding partner transcription factor when both are bound to the target site. Residue Ala47 lies within the first alpha helix of the HMG domain. It is adjacent to Asn46 which directly contacts DNA (Remenyi et al. 2003) and is within a cluster of amino acids that mediate the bending of the DNA strand (Scaffidi and Bianchi 2001). Residue His101 lies immediately C-terminal of the third alpha helical region but no specific role in the known HMG functions has been defined for this residue. Aspartic acid 123 (Asp123) lies outside the HMG domain in a region of unknown function but this residue is completely conserved within sequences from vertebrate species. The identical nucleotide change causing the p.(Asp123Gly) mutation here has been previously reported in a four-generation family with variable eye malformation (Mihelec et al. 2009). These are clearly independent events as the mutation in FamID 3227 has occurred de novo in the affected mother. The chromatograms and pedigrees for each of the intragenic mutations are shown in Figure S2.

#### Ocular phenotype

**Figure 2:**
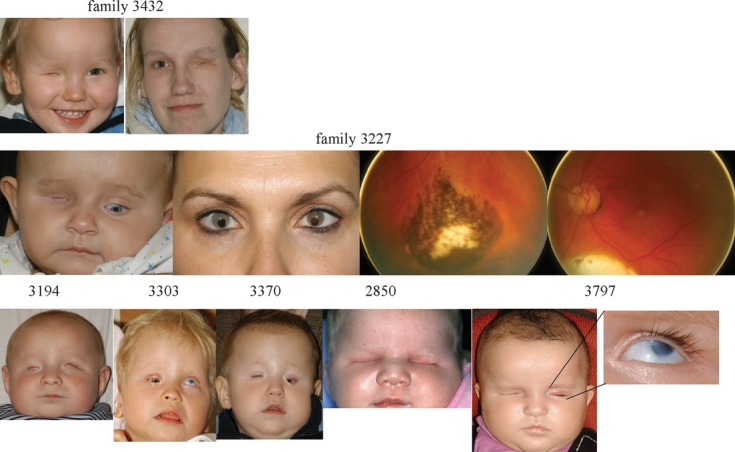
Portrait images of patients with a mutation in the *SOX2* gene. Affected son at age 3 years and his mother with unilateral anophthalmia at the right and left side (FamID 3432), respectively, are shown in the upper panel. The daughter with right anophthalmia, left microphthalmia, and microcornea at age 5 months and her mother with bilateral irido and left > right chorio-retinal coloboma are shown in the middle and fundus images in the left panel (CaseID 3327). Lower panel shows CaseID 3194 and CaseID 2850 at age 3 months and CaseID 3370 at 4 years (wears prosthetic shells) with bilateral anophthalmia, CaseID 3303 with right anophthalmia (wears prosthesis) and left microphthalmia, sclerocornea and microcornea at age 4 years, CaseID 3797 with right anophthalmia and left microphthalmia, sclerocornea and microcornea at age 3 months (small image).

Each of the 10 *SOX2* intragenic mutation cases reported here is associated with severe ocular malformation affecting at least one eye (Fig. 2). BA or UA with nonfunctioning microphthalmia (UA-UM) was present in four and two children, respectively. One of the children with UA-UM (CaseID 3303) did fixate light and objects as a small child but lost her vision due to sclerocornea and severe microcornea. Two families with transmission of a mutation from an affected mother to an affected child (FamID 3432 and 3227) were identified. In FamID 3432, the mother and son both had UA, on the left and right side, respectively. No ocular anomalies were seen in the other eye. In FamID 3227, the mother had bilateral iris and chorio-retinal coloboma with microcornea in the more affected left eye. Her first daughter showed a normal eye examination. Her second daughter was born with right anophthalmia and microphthalmia with microcrornea and chorio-retinal coloboma without fixation.

#### Extraocular phenotype and developmental data

The mean *Z*-scores for birth weight and birth occipitofrontal circumference (OFC) were 0.12 and −1.03, respectively. None of the birth weights were >2 SD from the population mean. Two affected individuals (CaseID 2813 and 3303) had congenital microcephaly (birth OFC *Z*-score ≤−2 SD). Postnatal head growth was significantly impaired with 6/8 cases having significant microcephaly. The mean *Z*-scores for postnatal OFC, height, and weight were −2.92, −0.85, and −1.16. Developmental follow-up information was available in 8/10 affected individuals: four and seven cases had delayed speech and motor development, respectively. None of the individuals with *SOX2* intragenic mutations show any significant extracranial malformations or abnormalities.

**Figure 3:**
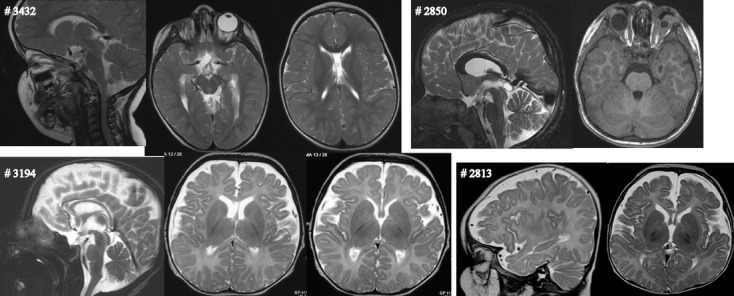
Cerebral abnormalities associated with a mutation in *SOX2* gene. Cavum vergae in a 3-year-old child with unilateral anophthalmia (CaseID 3432; proton-weighted sagittal and transversal spine echo 3 mm scan). Pineal cyst in a child with bilateral anophthalmia (note: orbital expander OU; CaseID 2850 at age 4.5 years; T2-weighted sagittal and T1-weighted transversal spine echo 3-mm scan). Small posterior part of the corpus callosum and septum pellucidum cyst in a child with bilateral anophthalmia (CaseID 3194 at age 3 months; T2-weighted sagittal and transversal spine echo 3-mm scan). Fronto-temporal cerebral volume reduction (T2-weighted native sagittal spine echo 2 mm and transversal 4-mm scan) in a 3-month-old boy with bilateral anophthalmia (CaseID 2813)

Brain MRI was available in 7/10 cases with intragenic mutations and one individual with a deletion. Of these eight cases, six had reported abnormalities on the scan. Four of eight cases had abnormal or dysmorphic midline structure such as hypoplasic posterior corpus callosum, septum pellucidum cyst, or cavum vergae. Fronto or fronto-temporal cerebral volume reduction but normally developed corpus callosum and midline structures were diagnosed in two of eight imaged children (Fig. 3).

### *OTX2* mutation cases

#### Consequence of mutations

Of the three different intragenic mutations in *OTX2*, two were plausible loss-of-function mutations, both deletions resulting in a frameshift within the ORF. The remaining change is a missense mutation with the potential to alter splicing, p.(Gln83His). Residue Gln83 lies within the homeodomain of OTX2 between the second and third alpha helical regions. This residue is conserved in all available arthropod orthologs, the orthodenticle protein (Table 4).

#### Ocular phenotype

**Figure 4:**
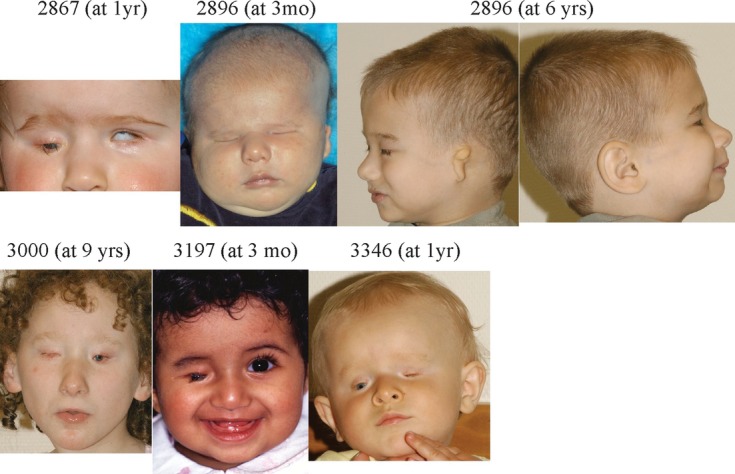
Portrait images of the children with mutations in the *OTX2* gene: CaseID 2867 at age 1 year with right anophthalmia (wears prosthetic shell) and left microphthalmia and microcornea. CaseID 2896 with bilateral anophthalmia and left microtia at age 3 months and age 6 years (wears prosthetic shells both sides; upper panel). CaseID 3000 at age 9 years with bilateral anophthalmia (wears prosthetic shell on the left side). CaseID 3197 with right anophthalmia at age 3 months. CaseID 3346 at age 1 year with bilateral anophthalmia (wears prosthetic shell on the right side).

Three of the four individuals with intragenic mutations of *OTX2* had severe malformations affecting at least one eye with two affected individuals showing complete blindness (Fig. 4). One of the affected children showed BA (CaseID 2896). CaseID 2867 has right anophthalmia and microphthalmia, microcornea and retinal coloboma in the left eye. The parents had observed some visual interaction in the first months of life, however, these were lost because of the severe ocular developmental anomalies. Her mother (CaseID 3362), who carries the same mutation, demonstrated a reduced vision due to amblyopia in the left eye but no coloboma or axial length abnormalities. Unfortunately, she did not consent for further investigations of retinal function (electroretinogram) or morphology (optical coherence tomography). CaseID 3197 had anophthalmia in the right but no ocular abnormalities in the left eye.

#### Extraocular phenotype and developmental data

There was significant postnatal failure of head growth. The mean *Z*-scores for birth weight and birth OFC were −0.56 and 0.02. The mean *Z*-scores for postnatal OFC, height, and weight were −3.2, −0.26, and −1.5. Two extraocular malformations were observed: CaseID 2896 has unilateral microtia (Fig. 3) and talipes equinovarus was observed in CaseID 2867. Developmental assessment revealed delayed speech and motor development in two of four children. CaseID 2896 is the most severely affected as he does not walk or crawl and has no receptive or expressive language skills. He has been fed by gastrostomy tube since 4 years of age. He has a very irregular sleep pattern and was treated with melatonin as a baby without success. His height and weight is below the third percentile.

Cerebral MRIs were available in three of the four affected individuals with *OTX2* intragenic mutations, with two of these showing no reported brain abnormality. CaseID 2896 showed no optic tracks but the brain was otherwise normal.

### *STRA6* mutation case

#### Consequence of mutation

CaseID 3279 has compound heterozygous mutations in *STRA6*, with both mutations predicted to result in complete loss of protein function. The exon 3 frameshift mutation c.120dupG p.(Pro41Alafs*39) is maternally inherited, and the exon 18 nonsense mutation c.1699C>T p.(Arg567*) is paternally inherited.

#### Ocular phenotype

This boy is blind. He has bilateral apparent anophthalmia with no cornea or sclera visible on clinical examination. However, a small eye is visible on MRI.

#### Extraocular phenotype and developmental data

He has normal motor development and age-appropriate speech and language skills. He attends a normal school. From a cardiorespiratory perspective, he has no chronic symptomatology, indeed he can participate in physically demanding sports. Following an episode of reactive airway disorder, he had a intensive pulmonary work up and this revealed a restrictive respiratory disorder. The pulmonary function test revealed reduced values (compared with age-matched normative data pre- and postphysical activity, respectively): total lung capacity (53%/68%), inspiratory vital capacity (51%/50%), forced expiratory volume after 1 sec (62%/57%), and maximal expiratory flow after 75%, 50%, and 25% of forced vital capacity.

**Figure 5:**
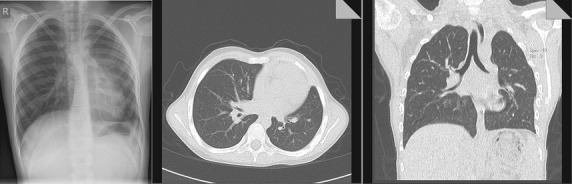
Chest X-ray (anterior–posterior scan) and chest CAT scan (axial and anterior–posterior scan) of CaseID 3279 with *STRA6* mutation show an elevated diaphragm, a right–left lung asymmetry with reduced lung volume in the left lung, and left-shifted heart.

The chest X-ray and computerized axial tomography (CAT) scan revealed an asymmetric lung with reduced volume in the left lung resulting in a left cardial cardiac shift (Fig. 5).

**Table 4:** Predicted consequences of identified missense mutations

Gene	Protein change	Align-GVGD class	PolyPhen-2 (score)	SIFT (score)	Mutation Taster (*P*-value)	SSF (scale 1–100, wt:mut [% diff])	MaxEnt (scale 1–12, wt:mut [% diff])	NNSPLICE (scale 0–1, wt:mut [% diff])	HSF (scale 1–100, wt:mut [% diff])
*SOX2*	p.(His101Arg)	C25	Probably damaging (0.996)	Deleterious (0.00)	Disease causing (1.0)				
*SOX2*	p.(Asp123Gly)	C65	Possibly damaging (0.616)	Deleterious (0.00)	Disease causing (1.0)				
*OTX2*	p.(Gln83His)	C15	Probably damaging (1.000)	Deleterious (0.00)	Disease causing (1.0)	84.9:72.3 (−14.9%)	9.5:2.2 (−77.2%)	0.75:0	89.7:78.9 (−12.1%)

wt:mut, the ratio of scores between wild-type and mutant alleles, respectively; % diff, the percentage difference between the wild-type and mutant allele score. Align-GVGD, align with Grantham variation (GV), Grantham deviation (GD); HSF, human splicing finder; MaxEnt, maximum entropy modeling of short sequence motifs; NNSPLICE, neural network splice site analysis; PolyPhen-2, polymorphism phenotyping v2; SIFT, sorting intolerant from tolerant; SSF, splicing sequences finder.

## Discussion

It has been recognized for several years that heterozygous loss-of-function mutations in *SOX2* or *OTX2*, mostly occurring de novo, are the major detectable genetic causes of severe human eye malformations. More recently bi-allelic mutations in *STRA6* have emerged as a rare but important cause of isolated anophthalmia in addition to the often lethal syndromal form. There have been many other studies that have analyzed single genes or combinations of genes in cohorts with various combinations of eye malformations (summarized in Table S1). In order to try to assess the relative contribution of each of the known anophthalmia/severe microphthalmia genes (listed in the Introduction), we analyzed a consecutive series of referrals to a single tertiary ophthalmology center. In this cohort, we have identified plausibly pathogenic mutations in the *SOX2*, *OTX2*, and *STRA6* genes which together account for 15/51 (29.4%) of the probands: 9/51 (17.6%), 5/51 (9.8%), and 1/51 (1.96%) for each gene, respectively. The proportions of affected individuals with *SOX2* or *OTX2* mutations are higher than those reported in previous studies (Wyatt et al. 2008; Schneider et al. 2009; Schilter et al. 2011), which probably reflects both the high percentage of patients at the most severe end of the ocular malformation spectrum in the present study and our inclusion of genome-wide aCGH to detect whole gene deletions as part of the mutation analysis. In total, 75% of bilateral and 20.7% of UA cases in our cohort are explained by mutations in these three genes.

Two examples of familial transmission of a *SOX2* mutation were identified. A *SOX2* missense mutation, c.368A>G p.(Asp123Gly), in one child with anophthalmia/microphthalmia that was inherited from her mother, who has bilateral chorio-retinal coloboma and microcornea in one eye. This mutation has arisen de novo in the mother. The mutation appears to be nonmosaic in the mother based on the observation of an equal ratio of mutant to nonmutant sequence on the chromatogram from blood DNA. We have, however, not been able to assess this ratio in other tissues of different embryonic origin. Remarkably, exactly the same mutation has been shown to segregate in a four-generation family of Australian descent, with a wide phenotypic spectrum ranging from iris hypoplasia to BA (Mihelec et al. 2009). There is no known function for this residue although it is located in a region of the protein that has been termed the partner factor domain (Kamachi et al. 1999). The second family is remarkable; both the mother and son have UA with a normal contralateral eye and both carry a frameshift mutation in *SOX2*, c.70_89del20 p.(Asn24Argfs*65), occurring before the sequence encoding the HMG domain. This mutation is apparently nonmosaic based on the mutant:nonmutant allele ratio in the sequencing chromatogram from blood DNA. The mutation has occurred de novo in the mother. This appears to represent the first known transmission of a nonmosaic complete loss-of-function allele in *SOX2*.

While familial recurrence of *SOX2* associated ocular malformation has been reported (Faivre et al. 2006; Schneider et al. 2008; Mihelec et al. 2009; Stark et al. 2011), these have been either missense mutations that may represent hypomorphic alleles or have been shown to be mosaic in the “carrier” mother. The first description identified a *SOX2* missense mutation in two children (anophthalmia/cryptophthalmos, severe hydrocephalus, and corpus callosum agenesis in a terminated fetus and BA in one sibling). Their unaffected mother was found to be mosaic for the same mutation resulting in this “pseudo recessive” pattern of inheritance (Faivre et al. 2006). Similarly, two daughters, anophthalmia and microphthalmia in one and BA in the other associated with partial agenesis of the corpus callosum, were born to a phenotypically normal mother who carries the same *SOX2* deletion in a mosaic state (Schneider et al. 2008). It is interesting to note that all transmissions of mutant *SOX2* alleles have been from the mother, which may suggest that female gametogenesis is more tolerant of reduced *SOX2* dosage than is spermatogenesis. This notion is supported by the finding of a reduction in male but not female fertility in mice that carry a heterozygous loss-of-function *SOX2* allele (Avilion et al. 2003).

Identifying nonocular malformations that are nonrandomly associated with mutations in “eye malformation” genes may give clues to the specific developmental mechanisms that are disrupted. In our study, we identified one individual with an *OTX2* mutation associated with unilateral microtia. Recently another unpublished child with bilateral microtia associated with BA has been identified in our laboratory (K. M. Girisha, unpubl. data), suggesting that this is a genuine association.

Intracerebral abnormalities are described in 30% of patients with anophthalmia-plus syndrome (reviewed in Makhoul et al. 2007). *SOX2* expression plays a crucial role in brain development, in particular the pituitary gland and hippocampal formation (Sisodiya et al. 2006; Kelberman et al. 2008; Alatzoglou et al. 2011). *Sox2* expression was identified in the forebrain, within Rathke's pouch and throughout the anterior but not posterior pituitary development, in the hypothalamus, and within neural ectoderm during human embryonic development (Kelberman et al. 2008), which becomes more restricted to the ventricular zone cells at midgestation (Sisodiya et al. 2006). Individuals with *SOX2* mutations are reported to have intracerebral anomalies including mesial-temporal malformation, hypoplasia or ectopia of the anterior pituitary gland or corpus callosum hypoplasia (Fantes et al. 2003; Ragge et al. 2005b; Sisodiya et al. 2006; Kelberman et al. 2008; Schneider et al. 2008). Alatzoglou et al. (2011) reported two children with *SOX2* HI, who developed slowly progressing hypothalamo-pituitary tumors.

Mutations in *SOX2* were associated with central nervous system (CNS) abnormalities outside the optic tract in all but two of the affected individuals on whom neuroimaging was available. Cerebral MRI scans had not been performed on three of the affected individuals, two of these were affected mothers. Most of the affected children showed midline anomalies such as cysts in the septum pellucidum or pineal gland, small corpus callosum or cavum vergae. Pituitary functional testing is not available from the children with *SOX2* mutations. Several children have growth measurement that map below the third percentile for height and or weight, indicating possible hormonal deficiency.

Mutations in *OTX2* are associated with pituitary structural abnormalities such as anterior hypoplastic and ectopic or absent posterior pituitary gland (Nolen et al. 2006; Diaczok et al. 2008; Tajima et al. 2009; Dateki et al. 2010; Schilter et al. 2011) and/or hormone deficiency (Nolen et al. 2006; Dateki et al. 2008, 2010; Diaczok et al. 2008; Tajima et al. 2009; Schilter et al. 2011). Partial or global growth hormone deficiency is the most common observed hormonal abnormality in patients with *OTX2* mutations. Other cerebral abnormalities associated with *OTX2* mutations are corpus callosum hypoplasia (Ragge et al. 2005a; Nolen et al. 2006), abnormal hippocampus (Ragge et al. 2005a; Dateki et al. 2008), ventriculomegaly (Ragge et al. 2005a; Nolen et al. 2006), or absent pineal gland (Henderson et al. 2007). One of the five children reported in our group showed a small pituitary gland. Cerebral MRI was performed in this child at age 4.7 years in comparison with the MRI scans performed between ages one day to 6 months in the other four children with *OTX2* mutations. Structural pituitary abnormalities in those children cannot be entirely ruled out because of the early age of imaging. Pituitary function is not available in our patient group. However, one child is below the third percentile for growth and weight, and two other children are below the third percentile weight. It is important that a high index of suspicion for functional pituitary anomalies is maintained for children with loss-of-function mutations in both *OTX2* and *SOX2*.

The absence of any intellectual disability and the relatively mild pulmonary phenotype in the boy with a compound heterozygous mutation in *STRA6* is interesting. This confirms that *STRA6* should be considered a rare but important cause of isolated ocular malformations in addition to the severe lethal multisystem disorders that were observed in the originally reported cases.

No mutations were identified in five of the eight genes screened: *PAX6* (paired domain), *BMP4*, *SMOC1*, *FOXE3*, and *RAX*. This may relate to the size of the cohort that was screened or, for the autosomal recessive loci (*SMOC1*, *FOXE3*, and *RAX*), population differences in disease allele frequencies and/or rates of consanguinity. While it is difficult to make firm conclusion, based on the published literature (summarized in Table S1) and our own unpublished data, *BMP4* and *RAX*, in particular, appear to be very rare causes of anophthalmia.

This study does have implications for the clinical molecular approach to the diagnosis of individuals with severe eye malformations. A screen for heterozygous loss-of-function mutations in either *SOX2* or *OTX2* should clearly be the first line test that will have a significant detection rate in cases of anophthalmia. It would also be reasonable to include *STRA6* in the initial analysis. However, different eye malformations are likely to require specific mutation screening strategies. None of these genes screened here represent significant causative loci for ocular coloboma, the single most common major eye malformation (D. R. FitzPatrick, unpubl. data). This may reflect genuine differences of the developmental pathology between these apparently overlapping classes of eye malformation but useful analysis must await the identification of the major causative loci for coloboma.

There remain many individuals in this cohort for whom we have not been able to identify a specific genetic cause. Whole genome and exome sequencing will be an important next step to identify new genes and/or genetic mechanisms that may account for this very interesting and important group of malformations.

## Supporting Information

**Figure S1.** Array CGH identified three heterozygous deletions encompassing the genes *OTX2* and *SOX2*. (A) Normalised log2 ratios indicating two deletions of *OTX2* in CaseID 3000 and CaseID 3346. (B) Normalised log2 ratios showing a deletion of *SOX2* in CaseID 2850.

**Figure S2.** Intragenic mutations identified in 12 families. A pedigree, mutation summary, and sequence chromatogram(s) are shown for each family with an identified intragenic mutation: eight *SOX2* mutation families, three *OTX2* mutation families and one *STRA6* mutation family. Excepting the parents in the recessive *STRA6* family, all individuals carrying a mutation are indicated by CaseID. For each pedigree all known affected individuals are shown, and all individuals shown were tested for the identified mutation. Unfilled symbols containing *N* represent unaffected individual with wild-type sequence; solid filled symbol, affected individual carrying mutation(s); dotted filled symbol, unaffected individual carrying mutation. All sequence chromatograms show the forward read, except for FamID 2867 where the reverse read is shown for clarity of data in a Gln (Q) repeat region. The wild-type reference sequence used for each gene is as follows: *SOX2*, NT_005612.15 GI:88966845; *OTX2*, NT_026437.11 GI:51493278; *STRA6*, NG_009207.1 GI:219275560.

**Table S1.** Reported studies.
