# Supplementary material for: Clinical and mutation analysis of 51 probands with anophthalmia and/or severe microphthalmia from a single center
Source: Mol Genet Genomic Med. 2013 Mar 27;1(1):15–31. doi: 10.1002/mgg3.2 (PMC3893155; doi:10.1002/mgg3.2)
Supplement: Supplementary file 1 [file mgg30001-0015-SD1.pdf]

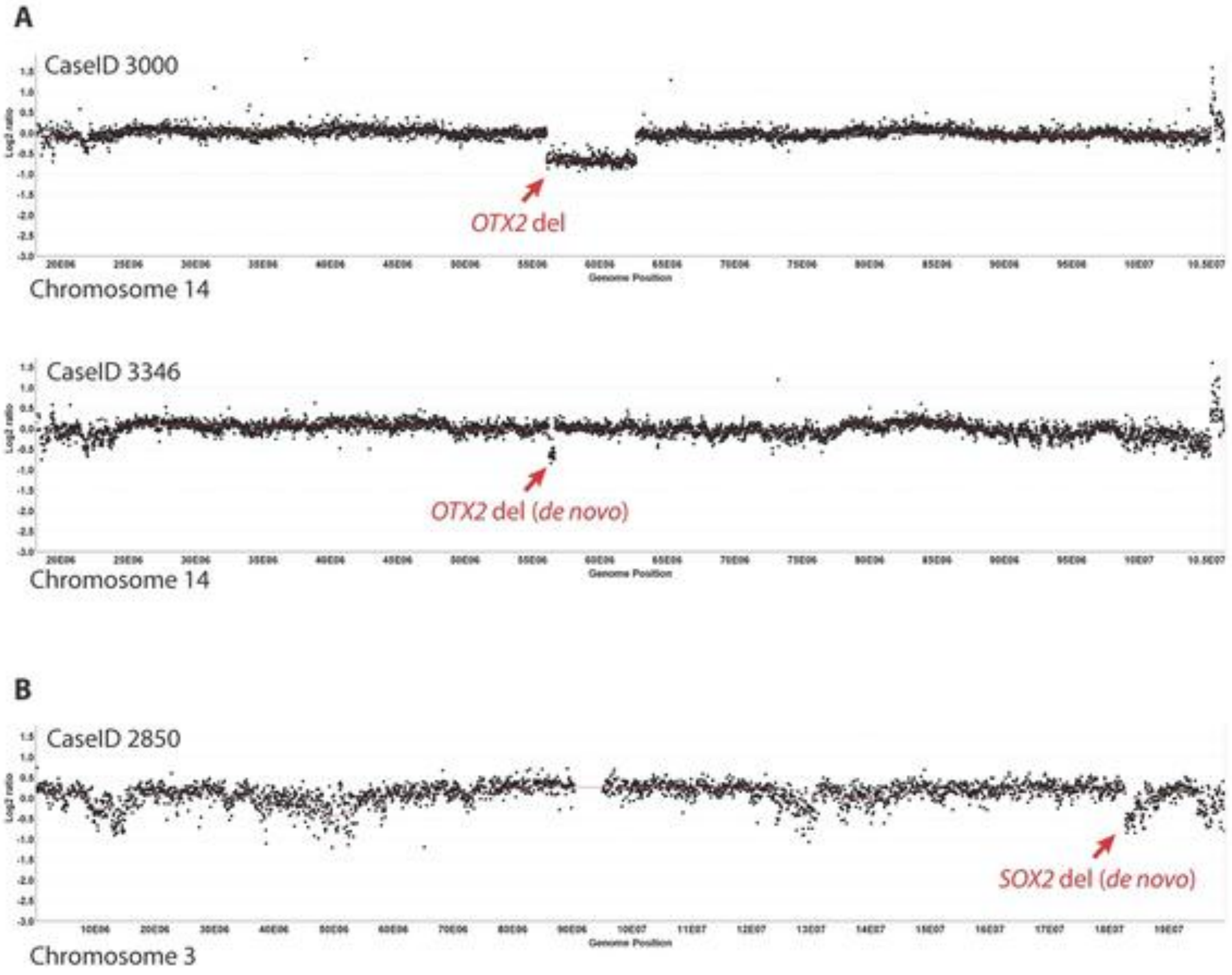

Supp. Figure S1. Array CGH identified three heterozygous deletions encompassing the genes *OTX2* and *SOX2*. **(A)** Normalised $\log_2$ ratios indicating two deletions of *OTX2* in CaseID 3000 and CaseID 3346. **(B)** Normalised $\log_2$ ratios showing a deletion of *SOX2* in CaseID 2850.

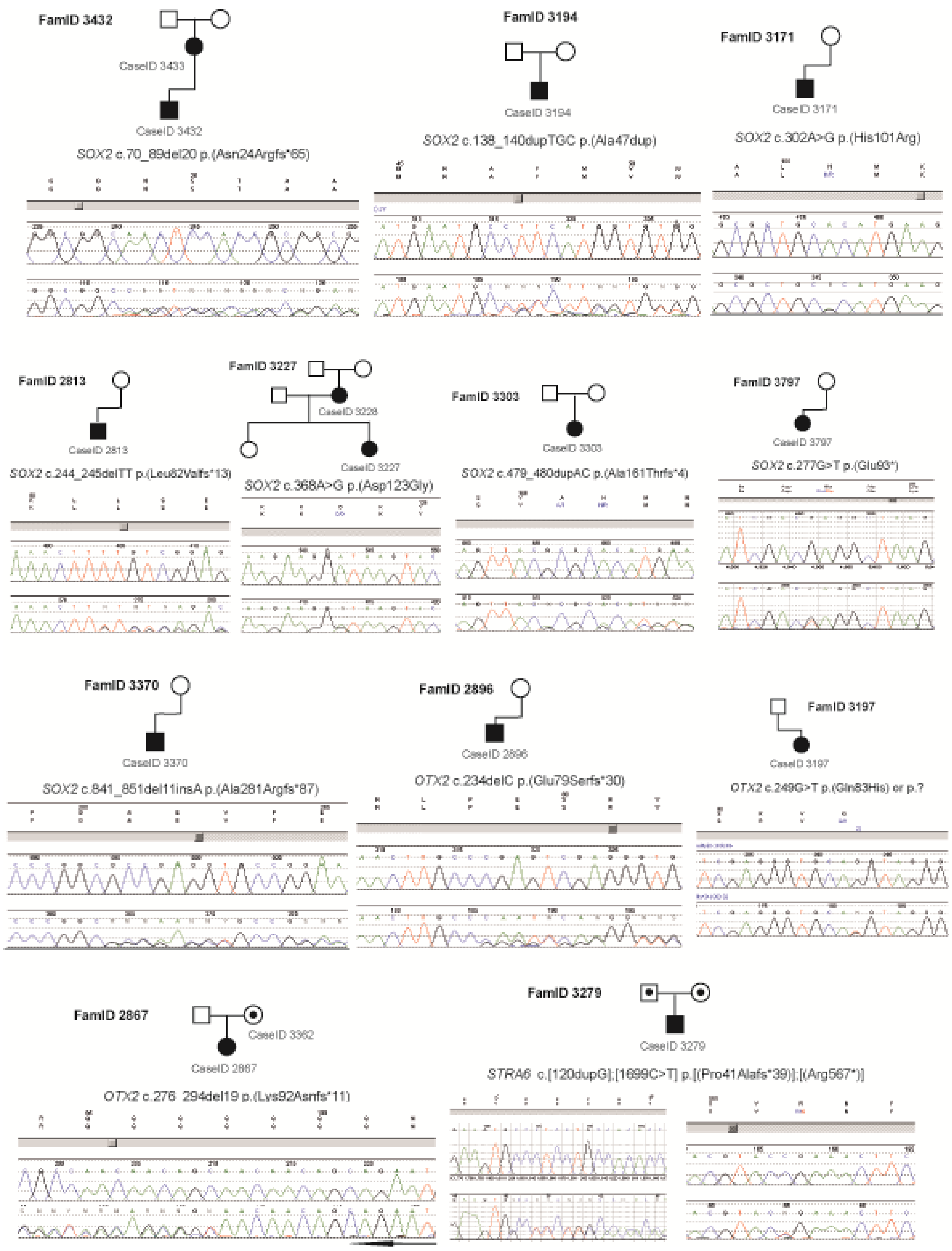

Supp. Figure S2. Intragenic mutations identified in 12 families. A pedigree, mutation summary and sequence chromatogram(s) are shown for each family with an identified intragenic mutation: eight *SOX2* mutation families, three *OTX2* mutation families and one *STRA6* mutation family. Excepting the parents in the recessive *STRA6* family, all individuals carrying a mutation are indicated by CaseID. For each pedigree all known affected individuals are shown, and all individuals shown were

tested for the identified mutation. Unfilled symbols containing N represent unaffected individual with wild type sequence; solid filled symbol, affected individual carrying mutation(s); dotted filled symbol, unaffected individual carrying mutation. All sequence chromatograms show the forward read, except for FamID 2867 where the reverse read is shown for clarity of data in a Gln (Q) repeat region. The wild type reference sequence used for each gene is as follows, *SOX2*, NT_005612.15 GI:88966845; *OTX2*, NT_026437.11 GI:51493278; *STRA6*, NG_009207.1 GI:219275560.
